# Cultural Variation in Emotional Reactivity and Regulation in Infants From Uganda and the UK

**DOI:** 10.1111/infa.70116

**Published:** 2026-08-19

**Authors:** Carlo Vreden, Eunice Ndyareeba, Herbert E. Ainamani, Rebecca Crowther, Helen Donnelly, Beatrice Forward, Brenda Kakai, Sarah Mazari, Josephine Paricia, Elizabeth Renner, John Sajabi, Katie Slocombe, Georgia Tuohy, Zanna Clay

**Affiliations:** ^1^ Department of Psychology Durham University Durham UK; ^2^ DIPF | Leibniz Institute for Research and Information in Education Frankfurt am Main Germany; ^3^ Department of Educational Psychology Kabale University Kabale Uganda; ^4^ Department of Mental Health Kabale University Kabale Uganda; ^5^ Budongo Conservation Field Station Masindi Uganda; ^6^ Department of Psychology Northumbria University Newcastle upon Tyne UK; ^7^ Department of Psychology University of York York UK

## Abstract

Learning to regulate one's emotions is a key feature of socio‐emotional development. The still‐face experiment—which measures how infants respond to a sudden change in maternal responsiveness—has long been considered a reliable indicator of infant reactivity and regulation. However, as a paradigm established to fit Western, middle‐class industrialized contexts, its robustness in other cultural contexts, has been questioned. Differences in responses to the still‐face experiment are likely shaped by variation in infant‐caregiver interactions and practices. To expand the literature and address sampling biases, we conducted the still‐face experiment with 10‐month‐old infants (*N* = 138, 68 female) across three sites, in rural and urban Uganda and urban UK. Across sites, we found the expected patterns of decreased infant positive affect and gaze toward their caregiver in the still‐face phase. However, while infants in urban Uganda and the UK exhibited increased negative affect when their mother ceased interacting, infants from rural Uganda did not. Our findings highlight important variation across socio‐cultural contexts in infant emotional reactivity and interaction style, suggesting that infants' responses to the still‐face paradigm may be shaped by the broader caregiving environments in which they develop.

## 1 Introduction

Emotion regulation, the ability to regulate one's emotions and behaviors effectively and appropriately in emotionally arousing situations, is key for navigating social interactions and has been linked to multiple socio‐emotional developmental outcomes, including positive social engagement, social competence, resilience, and empathy (Denham et al. 2003; Eisenberg and Fabes 1990; Eisenberg et al. 1997; Hein et al. 2018). Emotion regulation is proposed to first develop in the context of infant‐caregiver interactions: Initially, caregivers help to externally regulate infants' emotional arousal through soothing strategies, gradually scaffolding their ability to regulate their own arousal (Gergely and Watson 1999; Stern 1985). Over the course of the first year of life, infants begin to develop regulatory strategies, such as self‐soothing behaviors like thumb sucking, withdrawal from the situation, diverting attention or gaze (e.g., Braungart‐Rieker and Stifter 1996; Feldman 2009), whilst their regulation continues to be supported through caregiver interactions.

The still‐face paradigm (SFP; Tronick et al. 1978) has a long‐standing tradition in the study of infant emotion regulation development. In this seminal experiment, an infant and their caregiver, typically their mother, engage in a three‐phase interaction: (1) a *baseline* play phase, (2) a *still‐face* phase, where the adult ceases interaction and becomes unresponsive to re‐engagement attempts made by the infant, and (3) a *reunion* episode, resuming normal infant‐caregiver interaction. The “classic” still‐face effect is marked by a decrease in infant positive affect and gaze toward their caregiver along with an increase of negative affect from play to still‐face.

Like most classic behavioral paradigms in developmental research, the Still‐Face Paradigm was designed for and within the context of distal parenting, a form of parenting commonly found in North American and Western European industrialized middle‐class contexts that is characterized by high positive affect, contingent face‐to‐face interactions, eye gaze and verbal interactions (Kärtner et al. 2008, 2010). However, not only is this parenting strategy historically recent, but it is not representative of how most caregivers in the world parent, questioning its broader ecological and evolutionary relevance (Akhtar and Gernsbacher 2008; Field 2010). The Western‐centric bias embedded into the design and interpretation of the still‐face paradigm gives rise to two inter‐related concerns: First, behavioral responses to a paradigm designed within a specific cultural context risk being treat as a universal benchmark for “typical” or “healthy” development, which may undervalue alternative caregiving practices and the culturally‐specific developmental pathways they support. Second, in cases where the SFP *is* applied in alternative cultural/caregiver contexts, any divergences from the “classic” (i.e., Western) still‐face effect become difficult to interpret. This makes it difficult to differentiate whether potential variability arises from differences in infant emotion regulation, cultural divergences in how distress and engagement are expressed or the expectations infants have of their interactions with caregivers.

Thus, while the still‐face effect has been robustly demonstrated across many studies (see Mesman et al. 2009), most existing research on the SFP is biased toward a minority of the global population: namely Western middle‐class industrialized settings, mainly from North America and Western Europe, also termed “Minority world” populations (Alam 2008). A meta‐analysis of 39 still‐face studies (Mesman et al. 2009) included only one from a non‐Western population (i.e., China as compared to Canada; Kisilevsky et al. 1998). Moreover, only a limited number of studies have since replicated the still‐face effect in non‐Western settings (e.g., Gago‐Galvagno et al. 2022; Kisilevsky et al. 1998; Li et al. 2019; Owusu‐Ansah et al. 2019; Yato et al. 2008). This sampling bias is an increasingly recognized issue in developmental science as well as in the psychological sciences (Henrich et al. 2010; Nielsen et al. 2017) more generally. Most pertinent to development, sampling only a small and non‐representative minority of the world's population fails to account for global cultural diversity despite evidence highlighting the role that socio‐cultural settings play on social and cognitive development (Nielsen and Haun 2016). Even within Western settings, sampling is demographically biased toward urban, educated, middle‐class families, leading to reduced generalizability. Studies from non‐Western samples also typically follow the same demographic trend of sampling from urban, middle‐class families within post‐industrialized contexts where caregiving environments may be more comparable to Western settings.

Nevertheless, cultural variability may still be demonstrated: for example, 4‐month‐old Chinese and Dutch infants both demonstrated the expected change in positive affect and gaze, however, increased negative affect was less pronounced in Chinese infants (Li et al. 2019). In a Ghanaian sample, Owusu‐Ansah et al. (2019) found that six‐week‐old infants displayed the expected pattern in positive affect changes as Canadian infants but only if they had experienced high levels of everyday skin‐to‐skin contact, reflective of the enhanced role that skin‐to‐skin contact plays in parenting practices in this cultural setting.

In a more recent study, Broesch et al. (2022) addressed these demographic issues by comparing still‐face responses in a classic SFP as well as a culturally adapted SFP in three communities with different caregiving strategies: rural Bolivia, where caregivers practice high levels of physical contact (proximal care), Fiji and the US, where caregivers prioritize vocal and visual interaction (distal care). During their first year, infants in the US and Fiji displayed the classic still‐face effect but by comparison, the Bolivian infants did not. These effects were then replicated in a culturally adapted “Still‐Body‐Paradigm” conducted in Bolivia and the US, samples where the mother continued to hold the infant during the still‐face phase but stopped interacting otherwise, as an adaptation for caregiving settings which emphasize physical contact between infant and caregiver. The finding that rural Bolivian infants did not show the Still‐Face effect in either paradigm, as compared to infants experiencing distal care, emphasizes the effects of culturally specific caregiving practices on infant emotional development. Crucially however, the Bolivian infants showed evidence of increased tactile self‐stimulation, indicative of potential alternative regulation strategies that are not typically coded in the SFP. This suggests that infants in proximal care settings may respond differently in their regulatory and expressive behaviors to the SFP, which may not be captured by the standardized protocol which assess changes in affect or gaze. Conversely to Broesch et al. (2022), Wefers et al. (2023) compared responses to the classic SFP and to a no‐touch paradigm (where an experimenter first provided and then ceased physical stimulation to the infant) between infants from Germany and Ecuador and found an overall increase in negative affect during the no‐interaction phases of both paradigms. Taken together, these findings present a mixed picture of the construct‐validity of the SFP beyond Western settings: some culturally adapted paradigms replicate core aspects of the still‐face effect while others do not.

The comparability of studies in different socio‐cultural contexts is further complicated by methodological differences and study‐specific adaptations: Broesch et al. relied on mother‐infant interactions with varying levels of touch and facial affect, whereas Wefers et al. used experimenters and removed facial expressivity altogether. This makes it difficult to determine whether divergent findings reflect cultural variation or differences in what the tasks measure. These complexities highlight the broader challenge of balancing the application of paradigms not originally designed for certain caregiving styles, with the risk that extensive adaptations may even shift the underlying construct being assessed.

Therefore, it becomes especially important to consider how culturally grounded caregiving practices shape infants' expectations and therefore their responses to the classic, non‐adapted SFP. Differences in caregiving styles might lead to cross‐cultural variation in infant responses to the SFP in several ways: First, infants raised in proximal care settings might be less surprised by a break in social interaction than those regularly engaging in high levels of face‐to‐face communication (distal care), based on the expectations infants form about caregiver interactions from as young as 2 months (Bigelow and Rochat 2006). Second, infant emotional expressivity might be socialized differently across cultural contexts in accordance with culture‐specific socialization goals: Mothers from relational socio‐cultural contexts, where group harmony and interdependence are highly valued and proximal caregiving is common, are more likely to discourage the expression of high‐intensity emotions (e.g., Keller and Otto 2009; Lavelli et al. 2019). They may also suppress or dampen their emotional expressions when engaging with others, including with their child, in the interest of preserving interpersonal harmony (Cordaro et al. 2018; Friedlmeier et al. 2011). As such, infants of proximal caregivers may be less likely to express emotional arousal in the SFP due to socialized display rules.

Because the still‐face paradigm was designed within a cultural context that prioritizes face‐to‐face and positive affect interactions, it remains unclear to what extent the classic SFP response—that is, increased negative affect and decreased positive affect and gaze during the still‐face phase—can be expected from infants experiencing other types of caregiving, as found by Broesch et al. (2022) comparing infants from Fiji, Bolivia, and the US. To address these issues, the aim of the present study was to expand our understanding of the development of infant emotion regulation during the SFP by examining it in three cultural settings. Two non‐Western but related settings in Uganda—a rural and an urban site, respectively, along with a comparison group in a UK city.

British infants are known to be socialized according to Western models of caregiving and socialization goals, with a primarily distal parenting style (Keller and Otto 2009). By comparison, Ugandan infants typically receive proximal parenting, typical of non‐Western, subsistence farming populations. A recent study, conducted at the same rural Ugandan site as the present study, demonstrated evidence of proximal parenting, whereby the Ugandan infants had a higher number of caregivers, experienced more physical contact and were socialized toward interpersonal relatedness compared to the northeast UK, where caregiving styles were distal, with a socialization goal of independence and autonomy (Holden et al. 2022). Relatedly, differences in caregiver carrying style across the Ugandan and UK settings may also influence infant responses to the SFP, particularly with respect to face‐to‐face interactions. Like in many African countries, Ugandan infants are primarily carried on the back while mothers engage in daily chores (Mbada et al. 2019). Thus compared to UK infants, who engage in regular face‐to‐face interactions, facilitated through the use of pushchairs, carrycots and front‐facing baby carriers, Ugandan infants may have less opportunities and thus less expectations for face‐to‐face interaction as compared to UK infants.

Infant caregiving is shaped by a mosaic of cultural, demographic and socio‐economic factors and thus should not be generalized at the level of nationality. For instance, while mothers in our rural research site in Uganda showed the prototypical pattern of proximal parenting, the effects of urbanization in African settings, like Uganda are unknown. One possibility is that urbanization and exposure to Western media may result in more westernized caregiving practices. To examine this issue, we also sampled mothers and infants from an urban site in Uganda. Participant questionnaires, interviews and input from local researchers revealed numerous key differences between our rural and urban sites, including access to Western media, parental education, household structure, engagement in wage labor and caregiving styles (e.g., distributed caregiving, socialization goals). By comparing variation within a cultural setting, our study aimed to examine the influence of more nuanced socio‐cultural variation beyond highly contrasted comparisons.

Using the Still‐Face Paradigm, the aim of the current study was thus to examine how an infant's socio‐cultural context influences how they respond to an emotionally arousing caregiver interaction. We hypothesized that infants from the three sites would all respond to the SFP with decreased positive affect and gaze when the mother stops communicating with them. However, we expected cross‐site variation in infant negative affect, based on cultural variation in caregiving style and parenting attitudes (Holden et al. 2022). Specifically, as proximal caregiving styles in rural African settings like Uganda place less emphasis on face‐to‐face interactions (e.g., Gerlach et al. 2014; Holden et al. 2022; Kärtner et al. 2010), we predicted that infants in both of the Ugandan samples would be less distressed in the still‐face phase, as compared to the UK. We took a more exploratory approach in our comparison of urban and rural settings in Uganda, given previous research is lacking. On one hand, if urban Ugandan families are more exposed to westernized ideals and engage in more comparable urbanized lifestyles, we should expect less pronounced differences in the urban Ugandan site compared to the UK urban site. On the other hand, despite lifestyle differences, their caregiving practices and values may still be culturally conserved and thus their infants' responses more similar to the rural Ugandan site. This would potentially highlight the pervasiveness of culturally specific socialization strategies despite the growing influence of urbanization and globalization.

By testing if and how a well‐established paradigm like the SFP generalizes to more diverse and understudied contexts, we can both broaden our understanding of the factors shaping infant emotional development and use this knowledge to develop culturally appropriate adaptations of classic tasks.

## 2 Methods

### 2.1 Participants and Sites

In total, 138 infants and their mothers took part in the study, across two sites in rural and urban Uganda, as well as the UK. Because this study was part of a larger longitudinal cohort of mother‐infant dyads who were followed throughout the first 2 years of life, no power analysis for this specific study was conducted. Data collection took place at a central research location at each site (on a university campus in the UK, a college campus in rural Uganda, and in a primary school in urban Uganda). All rooms were minimally furnished, avoiding distracting objects or toys, and were in a part of the building with few noise distractions. At each site, data collection was led by local researchers who spoke the mother's native language.

Below, we overview the site characteristics which together, reflect three distinct socio‐cultural caregiving environments. Although extensive demographic and contextual information was collected (e.g., maternal education, household income, media exposure), these variables were strongly structured by site and were not measured on directly comparable scales across contexts. Rather than representing independent covariates, they reflect interconnected features of the broader caregiving ecologies that distinguish the study sites. We therefore report these variables descriptively to characterize the participating populations but do not treat them as separable predictors in the primary analyses.

#### 2.1.1 Rural Uganda

Forty‐seven infants (22 female, 25 male) participated (*M*_age_ = 48.77 weeks (SD = 3.04)) in rural Uganda. The research site consists of multiple villages located in Nyabyeya Parish, Masindi district in Western Uganda. Participants were recruited via word of mouth by local researchers in villages in the area. According to our background surveys, most participating mothers had limited literacy, mostly incomplete primary school education, and family income (on average £30 per month in our sample) was largely from subsistence farming. Infant care was often distributed between multiple members of the household, including other children, who carry the infant on their back while engaging in daily chores. Media access was low, with 81% reporting they had no access to internet, and 60% reporting they had no regular access to TV. Families lived in multi‐generational (on average 5.48 people) family compounds consisting of multiple mud‐brick buildings, and most had at least one other child living in the same household as the participating infant.

#### 2.1.2 Urban Uganda

Fifty infants (25 female, 25 male) participated (*M*_age_ = 46.86 weeks (SD = 2.92)) at the urban Ugandan site in Mbarara, one of Uganda's largest cities. Recruitment was organized with the help of local village health teams and local researchers. Background questionnaires revealed that most mothers at the urban Ugandan site had completed primary school education and some secondary education. Engagement in wage labor was more common and monthly household incomes were roughly £100. Mothers' engagement in wage labor was more common in the urban than the rural sample. Furthermore, media exposure was higher than that reported in the rural sample, with 33% of mothers reporting access to internet and 73% regularly watching TV. With better access to education for other children in the household and more mothers working outside the home, caregiving was more likely to be distributed between adult household members than in the rural sample, where nonmaternal caregivers tended to be other children. Most families lived in single‐household homes (average household size 4.28 people), and most had at least one other child besides the participant living in the household.

#### 2.1.3 UK

Forty‐one infants (21 female, 20 male) participated (Mage = 47.12 weeks (SD = 2.57)) participated in the UK. Participants from in and around a small city were recruited through social media adverts and local baby groups. Most mothers had some form of tertiary education and were formally employed (average monthly household income at least £1250, with more than half of the sample earning at least £3750). 100% of mothers reported having internet access and regularly watching television. Childcare was distributed between parents, with some assistance from family members such as grandparents, in addition to nurseries. Households consisted of on average 3.69 people and about half of the sample had another child living in the same household as the participant.

### 2.2 Ethics

Ethical approvals were obtained from the Department of Psychology at Durham University, the Ugandan Virus Research Institute (GC/127/20/08/788), and the Ugandan National Council for Science and Technology (SS596ES). Information about the study was given by a local researcher in either written or verbal form, depending on mothers' level of literacy. In Uganda, the consent procedure was delivered by local researchers in the mothers' native language. Mothers who could not read or write gave their written consent as a thumbprint, an accepted form of signature in Uganda.

### 2.3 Materials

The task was filmed from two angles, one focusing on the infant and the other on the mother. In both cases, the whole body was filmed but at an angle where the face was visible. We used Panasonic HC‐VX870 4K camcorders with external microphones (Sennheiser MKE 400 Shotgun Microphone). One camera, focused on the mother, was mounted on a tripod; the other camera was handheld to account for infant movement. Infants were placed in a standard stroller with wheels fixed to stay in one place (see procedure). Any infants who did not tolerate the stroller were tested sitting on a playmat. Although a deviation from the classic set‐up, it was necessary to make some cultural adaptations given that strollers/car seats or other restraining devices are almost unheard of in Uganda, with infants primarily held or seated on the ground most of the time. A piloting phase established that being seated on a playmat served as the most culturally appropriate alternative. We statistically controlled for infant position in our analyses.

### 2.4 Procedure

The Still‐Face Paradigm (SFP) consists of three phases: play, still‐face, and reunion. In the *play* phase (2 minutes), the mother was instructed to play with her child as she normally would, without the use of objects. Mothers were allowed to touch and engage as they wished with their child. If this led to the mother touching or holding the infant at the end of the play phase, they were instructed to place them back down on the mat and out of physical contact at the end of the play phase. This was then followed by the *still‐face* phase (1 minute), whereby the mother was instructed to stop interacting with the infant, look at the infant's forehead (but not at their eyes) with a neutral expression, not touch the infant or respond to any attempts by the infant to reengage her attention. In the *reunion* phase (2 minutes), mothers were instructed to play with their child again, as they normally would and could also touch them again (again without objects). A researcher timed each phase and verbally instructed the mother when to move onto the next phase. The researcher stood behind the infant and used minimal instructions (e.g., “And still‐face!”). If the mother broke character during the still‐face, the trial was abandoned and repeated on the same day with a break and other tasks in between.

Infants were seated in the stroller facing their mother who was sitting on a chair. Infants were provided a habituation phase to acclimatize to the stroller while other questionnaires for a larger study were undertaken. As noted above, any infants who did not tolerate the stroller completed the SFP seated on the mat in front of their mother (40% at the rural Ugandan site, 56% at the urban Ugandan site, 10% in the UK). In this case, the mother was seated ca. 50 cm away from the infant also on the mat but was instructed similarly to the stroller‐version not to touch her child.

### 2.5 Coding

Behavioral coding was completed using ELAN, an open‐source audio and video annotation tool (Wittenburg et al. 2006). We coded three separate scales of infant behavior—positive affect, negative affect, and gaze—based on Li et al. (2019). Positive affect was based on positive facial expressions, for example, smiling and vocalizations, such as giggling or laughing. Negative affect was coded based on negative facial expressions, such as frowning, and vocalizations, including whimpering and crying. Affect was rated on a 0–3 scale of no (0), low (1), medium (2), and high (3) positive/negative affect. We coded the duration of infant gaze at their mother, based on eye gaze direction visible in the infant‐focused camera angle. Gaze was also coded on a 0–3 scale of no gaze toward the mother (0), gaze toward the mother for less than half of the coded Section (1), more than half (2), and all of the coded Section (3), with the prerequisite that the infant's face was visible for at least two thirds of the coded section. The full coding scheme is available in the supplementary information (Supporting Information S1: Table S2). Infants received one rating of 0–3 per phase (play, still‐face, reunion) and behavior (positive affect, negative affect, gaze).

#### 2.5.1 Inter‐Observer Reliability

Behavioral coding was split between three coders who completed inter‐rater reliability for 20% of all videos. Reliability was good with Cohen's kappa at 0.74–0.81 for positive affect, 0.71–0.78 for negative affect, and 0.78–0.92 for gaze.

### 2.6 Statistical Analysis

Statistical analyses were conducted using R (version 4.2.2; R Core Team 2020). Given the ordinal nature of our rating data, we used cumulative link mixed models (CLMM) with the *ordinal* package (Christensen 2023). We first established model significance by comparing each full model containing all predictors of interest to a null model dropping the predictors of interest but retaining control fixed effects and, where applicable, random effects, using a likelihood‐ratio test (Dobson and Barnett 2008). This was followed by a second likelihood‐ratio test between the full model and a main effects model (into which interaction terms from the full model were instead entered as independent main effects). Post‐hoc tests were run using the emmeans package, with Tukey's adjustment for multiple comparisons (Lenth 2021). We constructed three separate CLMMs to test variation across sites in infant expression of positive affect, negative affect, and gaze during the SFP, with each behavioral measure as the outcome variable. These models further included an interaction of experimental phase (play, still‐face) and site (rural Uganda, urban Uganda, UK), controlling for testing set‐up (mat, stroller), and with a random intercept for participant ID.

Our analysis focussed only on the change from play to still‐face phase (not including the reunion phase), as the existing literature suggests this to be the most robust (e.g., Mesman et al. 2009) and thus most likely to replicate cross‐culturally. Although the reunion phase can provide insights into caregiver‐mediated regulation and recovery, responses during reunion are necessarily contingent on infants' reactions to the still‐face episode itself. Anticipating cross‐site differences in infant responses to the still‐face phase, we omitted an analysis of reunion responses to avoid conflation of regulatory recovery with preceding differences in reactivity.

Finally, although demographic and contextual information was collected (as noted), these were not included as predictors, as they strongly clustered by site and are thus better understood as descriptive indicators of broader caregiving ecologies rather than individual‐level predictors. As shown in Supporting Information S1: Table S1, sites differed systematically across multiple interconnected dimensions, including maternal education, household income, media access, household composition, employment, and caregiving arrangements. These variables were collected to characterize the caregiving environments in which infants were embedded and, in several cases, were not measured on directly comparable scales across sites (e.g., household income). Consequently, they do not readily lend themselves to interpretation as equivalent individual‐level covariates. Rather, they reflect interrelated features of the broader socio‐cultural contexts that distinguish the study sites. Including them as covariates would therefore risk statistical overcontrol by partialling out meaningful aspects of the caregiving ecologies under investigation, while also complicating interpretation due to their strong association with site. Accordingly, these variables are reported descriptively to provide contextual information for interpreting site differences but were not included in the primary models.

Full model estimates can be found in the supplementary materials (Supporting Information S1: Tables S3.1–S3.3). All relevant data and analysis code are available on the Open Science Framework: https://osf.io/4chvy/?view_only=a4f3feb698084f6bbaf058ae28c4f0a5.

## 3 Results

### 3.1 Positive Affect

**FIGURE 1:**
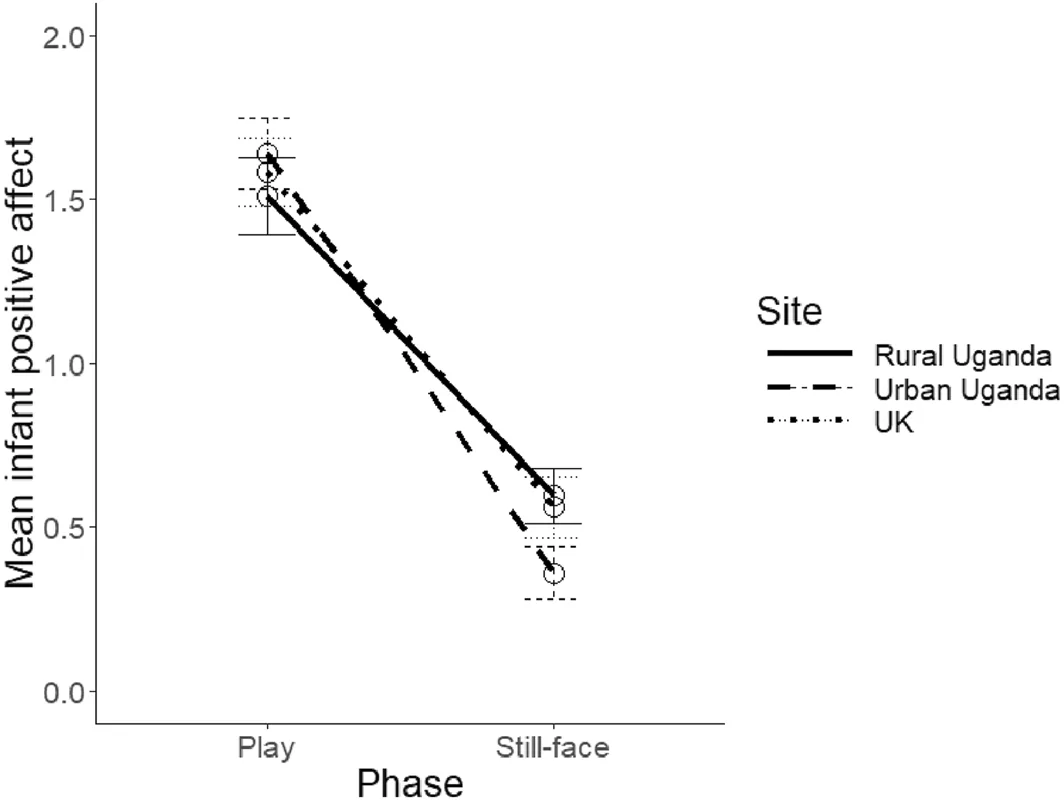
Mean infant positive facial affect during the still‐face paradigm by experimental phase and site.

The full‐null model likelihood‐ratio test for positive affect was significant (*χ* ^2^(5) = 143.31, *p* < 0.001) but the full‐reduced model comparison was not, suggesting no interaction between phase and site (*χ* ^2^(2) = 5.99, *p* = 0.050). Continuing with the reduced model, as predicted, there was a main effect of phase with infants across sites showing significantly lower positive affect during the still‐face than the play phase (*χ* ^2^(1) = 136.87, estimate (SE) = −3.61 (0.43), *p* < 0.001, see Figure 1). There was no main effect of site (*χ* ^2^(2) = 0.47, *p* = 0.791).

### 3.2 Negative Affect

**FIGURE 2:**
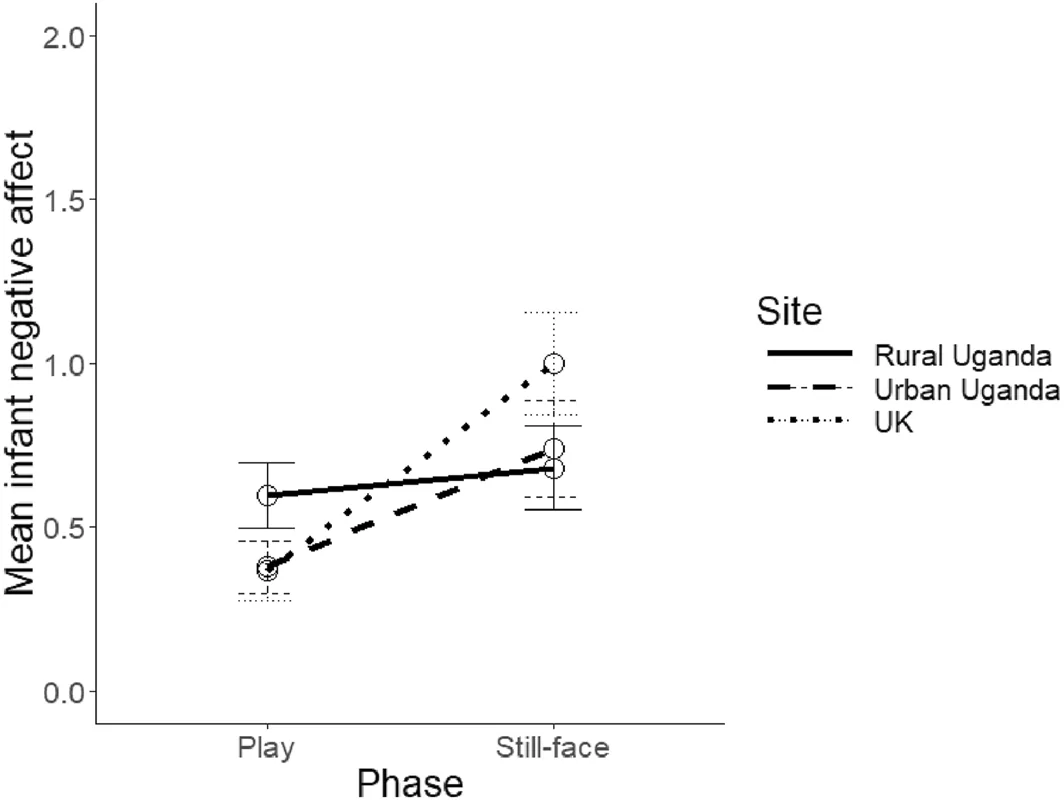
Mean infant negative facial affect during the still‐face paradigm by experimental phase and site. Affect coded on a 0–3 scale of overall intensity. Error bars represent the standard error of the mean.

The full‐null model likelihood‐ratio test for negative affect was significant (*χ* ^2^(5) = 21.14, *p* < 0.001), as was the full‐reduced model comparison (*χ* ^2^(2) = 7.95, *p* = 0.019), suggesting significant site variation in infant negative affect according to phase, as predicted. Post‐hoc tests showed that infants in the UK (estimate (SE) = 2.07 (0.54), *p* < 0.001) and in urban Uganda (estimate (SE) = 1.07 (0.49), *p* = 0.031) showed increased negative affect during the still‐face phase, but infants from rural Uganda did not (estimate (SE) = 0.17 (0.45), *p* = 0.697). See Figure 2. Testing setup, a control variable, showed a significant main effect on negative affect (estimate (SE) = −1.21 (0.49), *p* = 0.012), indicating overall differences in negative affect between infants tested on a mat versus in a stroller or seat, averaged across interaction phases.

### 3.3 Gaze

**FIGURE 3:**
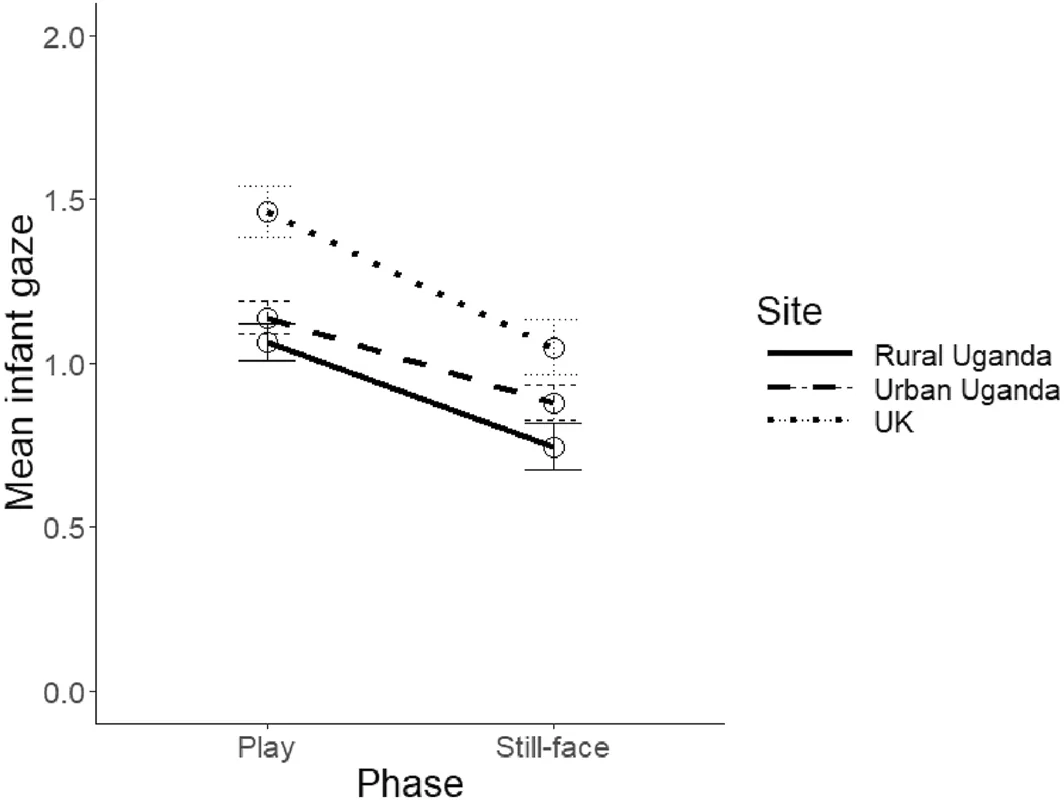
Mean infant gaze during the still‐face paradigm by phase and site. Gaze coded on a 0–3 scale of relative occurrence. Error bars represent the standard error of the mean.

The full‐null model likelihood‐ratio test for gaze was significant (*χ* ^2^(5) = 106.93, *p* < 0.001), as was the full‐reduced model comparison (*χ* ^2^(2) = 14.37, *p* < 0.001), suggesting significant site variation in infant gaze according to phase. Post‐hoc tests showed that infants in the UK showed higher levels of gaze toward their mother compared to rural and urban Ugandan infants in the play phase (rural Uganda vs. UK: estimate (SE) = 11.57 (2.81), *p* < 0.001; urban Uganda versus UK: estimate (SE) = 10.31 (1.71), *p* < 0.001) and in the still‐face phase (rural Uganda vs. UK: estimate (SE) = 3.85 (1.42), *p* = 0.019; urban Uganda versus UK: estimate (SE) = 3.73 (1.18), *p* = 0.005). See Figure 3.

## 4 Discussion

Emotion regulation is a key developmental process linked to a variety of socio‐emotional outcomes (e.g., Eisenberg and Fabes 1990; Eisenberg et al. 1997). Nevertheless, it is unclear whether the main paradigm used to study its development in infancy, the still‐face paradigm (Tronick et al. 1978), is robust across more diverse cultural settings. The still‐face paradigm was developed for and within minority‐world Western contexts, namely North America and Europe, which represent caregiving settings that favor high positive affect, contingent face‐to‐face interactions, eye gaze and verbal interactions. Importantly, such contexts are unrepresentative of most infants' caregiving experiences worldwide. In the present study, we addressed this by examining infant responses to the classic still‐face in two related but distinct Majority‐world caregiver settings: urban and rural Uganda, as well as in the UK. By diversifying beyond standard Western samples, our aim was to broaden our understanding of global infant socio‐emotional development and gain novel insights for the development of culturally appropriate developmental paradigms.

As expected, we replicated the classic still‐face effect (Mesman et al. 2009) for positive affect and gaze across all three sites: infants showed decreased positive affect and gaze toward their mother during the still‐face phase as compared to the play phase. This suggests that across sites, infants' positive emotional communication is stimulated by emotional cues offered by their caregiver, while the meaning of gaze engagement may be more context‐dependent and shaped by differences in everyday interactional experiences. Crucially, our results showed that gaze engagement differed substantially at baseline across sites, with infants in both Ugandan samples showing significantly lower levels of gaze toward their mother than UK infants during the initial play phase. This site difference in infant gaze suggests that reductions in gaze during the still‐face phase may not carry equivalent psychological meaning across sites. In caregiving contexts, such as Uganda, where sustained face‐to‐face interaction and direct eye contact between infant and mother may be less central to everyday caregiving, gaze may reflect contextually shaped norms of attention and interaction rather than emotional distress or disengagement per se.

By comparison, we found greater site variation for infant negative affect. Interestingly, while infants from both urban UK and urban Uganda showed increased negative affect when their mother stopped interacting with them, infants from rural Uganda did not show a strong response. These results reflect findings by Broesch et al. (2022), who found that rural Bolivian infants, exposed to high levels of proximal care, which is similar to infants from rural Uganda (Holden et al. 2022), did not show increased negative affect when their mother stopped interacting with them in both the classic Still‐Face paradigm as well as a culturally‐adapted Still‐Body paradigm where mother and infant remained in physical contact (but interaction ceased otherwise). From as young as 2 months old (Bigelow and Rochat 2006), infants form expectations about social interaction. Thus, infants raised in caregiving settings such as the UK, where face‐to‐face interactions are prioritized, may expect high levels of face‐to‐face interactions and thus react more negatively when these expectations are violated. Infants from proximal care settings, such as rural Uganda, may not hold the same expectations and thus be less affected by a cessation of this interaction (note that descriptively there still was a tendency for increased negative affect in all sites). Together, these findings suggest that while some components of the still‐face response, namely positive affect, may generalize across the caregiving contexts examined here, others—particularly gaze—may index different processes which depend on the infant's developing expectations about caregiver interactions.

In our study, infants rural Uganda, a proximal care setting, showed smaller increases in negative affect in response to the still‐face phase, but interpreting whether this reflects differences in underlying emotion regulation, reactivity, or culturally shaped expression styles is complex. Evidence suggests that infants who experience higher levels of proximal care are less likely to display negative emotions in general, including in urbanized Western contexts (e.g., Hunziker and Barr 1986; St James‐Roberts et al. 2006). One proposed explanation is that proximal caregiving, which prioritizes close contact, equips infants with different, possibly more effective, emotion regulation strategies. For example, in a study comparing frustration responses of rural South African infants (proximal care) and urban UK infants (distal care), rural South African infants spent 10 times as much time exhibiting self‐soothing behaviors than UK infants (Bozicevic et al. 2016). In our study, support for this hypothesis could not be easily differentiated from differences in reactivity as infants from rural Uganda appeared to find the still‐face event less distressing in the first place. Future research is needed to systematically examine whether variation in caregiving practices (e.g., level of face‐to‐face engagement and tactile contact) both within and between socio‐cultural settings predicts differences in infant emotional reactivity, regulation and the interaction between them. This would benefit from incorporating physiological measures to assess if observed differences in infant behavior are due to lower reactivity, indicated by lower physiological arousal, or greater regulation effectiveness, indicated by use of regulation behaviors and sharper return to baseline.

By including a more nuanced within‐country comparison between rural and urban settings within a non‐Western setting, our study went beyond a stark cross‐cultural approach of West/non‐West comparisons. Our goal was to examine whether broader socio‐economic changes in relation to urbanization and globalization within a majority‐world context like Uganda may lead to a closer alignment toward more “Westernization” caregiving practices and developmental trajectories. We found some evidence for this in respect to displays of negative affect, with urban Ugandan infants showing a more similar pattern than rural Ugandan infants as those from the UK. However, because the sites differed across multiple interconnected demographic, material, and caregiving characteristics, the present design cannot determine which specific features of the caregiving environment contributed to these similarities and differences. While the rural setting we sampled in Uganda has been demonstrated as proximal, as is typical of subsistence farming populations (Holden et al. 2022), we did not conduct a systematic, interaction‐level assessment of caregiving practices at the urban Ugandan site (e.g., face‐to‐face engagement, tactile soothing, or contingent responsiveness), and thus no such fine‐grained data are available for the urban Ugandan infants. Therefore, we cannot say with certainty what specific differences in caregiving practices at the two Ugandan sites lead to the observed differences in infant emotional reactivity. Any inference that urban Ugandan caregiving converges toward Western‐style distal practices should therefore be treated cautiously. Nevertheless, emerging evidence supports meaningful cross‐cultural variation in caregiving responses to infant distress; for example, Vreden et al. (2025) found that rural Ugandan mothers relied more heavily on physical soothing than UK mothers, consistent with proximal caregiving models (although as above, no such work is currently available for urban Uganda). A systematic examination of caregiving practices at these sites and how these are associated with infant emotional responses is needed to address what aspects of emotional socialization and lifestyle shape infant outcomes. e.g., naturalistic observations could be used to quantify infants' everyday exposure to face‐to‐face interactions, physical contact, and distributed caregiving. These measures could then be linked to infants' affective, attentional, and regulatory responses during the still‐face episode. Additionally, experimental manipulations of the still‐face paradigm itself (e.g., varying the amount of gaze, touch, or vocalization, as has been done by Broesch et al. in the still‐body paradigm) would allow stronger inference about how culture‐specific caregiving experiences shape infant responses. Such approaches would help disentangle whether observed site differences reflect variation in emotional reactivity, regulation, caregiving practices or other features of infants' developmental environments.

Despite these contributions, several limitations should be acknowledged. As noted in earlier sections, the still‐face paradigm itself may not capture all relevant dimensions of emotion regulation, especially in cultures where face‐to‐face interaction is less central. This limitation may be especially relevant for the gaze measure. Infants in both Ugandan samples showed substantially lower levels of gaze toward their mother already during the play phase, prior to the onset of the still‐face. As such, a reduction in gaze during the still‐face phase may not reflect the same emotional or regulatory processes across cultural contexts. Gaze declines may still reflect emotional distress or regulation but should also be interpreted in the context of culturally normative patterns of attention and engagement.

An additional limitation concerns the interpretation of site differences. As shown in Supporting Information S1: Table S1, sites differed systematically across multiple demographic and contextual characteristics, including maternal education, household income, media access, household composition, employment, and caregiving arrangements. These factors were strongly patterned by site and were collected using measures designed to be locally appropriate rather than directly comparable across settings. Consequently, the present study cannot determine whether observed site differences are attributable to any specific cultural, demographic, or environmental factor in isolation. Rather, they should be interpreted as reflecting variation across broader socio‐cultural caregiving ecologies. Future research that directly measures caregiving practices and employs comparable contextual measures across sites will be necessary to identify the mechanisms underlying these differences.

Further, to culturally adapt the SFP in a context where infant seats or strollers are rare, infants who did not tolerate the standard experimental setup were tested on a playmat. Being tested on a mat instead of a stroller was linked with infants' overall negative affect, but not their positive affect or gaze. To account for this, testing setup was included as a control variable in our analyses. We found that testing setup had a significant effect on infant responses across phases, suggesting it can influence overall patterns in affective responding rather than specifically during the still‐face episode. This highlights the importance of future work to consider physical context, especially when comparing infant behavior across sites. As our research shows, conducting cross‐cultural research, especially with infants and young children, is a careful balance between maintaining ecological and construct validity and methodological rigidity/flexibility (Wen et al. 2025). In the case of the still‐face paradigm, prior cross‐cultural research has shown that response patterns seem to remain stable, even with methodological adaptations (Broesch et al. 2022), however, we acknowledge that any change to the “classic” still‐face experiment constrains direct comparability with previous studies.

Lastly, we focussed on the play‐still‐face comparison, which is the most robust element of the SFP across studies, we acknowledge that excluding the reunion phase limits comparability with studies that analyze all three phases of the SFP. The reason for this was grounded in both methodological and conceptual considerations. Given it is the last phase in the interaction, the reunion behavior reflects a complex interplay between infant reactivity to the still‐face phase and ensuing caregiver regulatory behavior during the reunion phase, both of which may vary not only on an individual basis but also systematically across cultural settings. In the present study, differences in infants' responses to the still‐face phase, combined with variation in testing setup (e.g., mat/stroller), would have made it difficult to disentangle culturally meaningful differences in recovery from artifacts of preceding distress or physical context. Future work using more standardized setups or modeling phase‐by‐phase dependencies could more directly address cross‐cultural variation in reunion dynamics.

Taken together, our study helps disentangle two key issues in the construct‐validity of the still‐face paradigm and conducting cross‐cultural research on infant regulation: the risk of treating patterns observed in Western infants as a universal developmental benchmark for infant emotionality, and the validity of applying the still‐face paradigm across diverse caregiving ecologies where responses and caregiving styles differ from those to which it was designed. On the one hand, we replicated the classic still‐face effect for positive affect across all three sites, suggesting some degree of robustness across diverse caregiving contexts in measuring infants' sensitivity to caregiver unavailability. On the other hand, we observed site‐specific patterns for infant negative affect and gaze, with infants from rural Uganda showing no significant increase in negative affect and both Ugandan samples exhibiting substantially lower baseline gaze than suburban UK infants. These differences should not be interpreted as evidence for the influence of culture in isolation, but rather as reflecting variation across broader socio‐cultural caregiving environments. Overall, these findings demonstrate that the need to for caution when seeking to identify Western‐centric patterns of still‐face responses in non‐Western proximal caregiving settings where they may not be appropriate or typically. Failing to do so could result in typical developmental patterns and caregiving practises being misunderstood or even misrepresented. From a construct‐validity perspective, baseline differences in gaze engagement and attenuated negative‐affect responses in proximal‐care settings illustrate that the classic SFP can reliable measure culturally‐shaped expectations about expressivity and interaction, at least as much, if not more, than it is a measure of emotional reactivity and regulation.

Over 4 decades of research has supported the validity of the SFP as a reliable measure of infant regulation (Mesman et al. 2009). Nevertheless, most of this research has been derived from participant samples in minority‐world contexts, whereas its ecological validity in more diverse settings is less understood. By replicating the classic still face effect for positive affect in two more diverse settings in Majority‐world contexts, one rural and one urban, our study provides partial support for its global validity. Nevertheless, we did find variation in expression of negative affect across sites, which may be related to differences in caregiving environments and infants' everyday social experiences. In summary, our study contributes to an emerging body of research (e.g., Broesch et al. 2022; Li et al. 2019) which highlights cross‐cultural continuity as well as variation in response to the still‐face paradigm. Our findings highlight both robust and context‐sensitive aspects of infant responses to the still‐face paradigm. Future research should carefully consider the development of more context‐sensitive adaptations of the Still Face paradigm when studying non‐Western caregiving contexts to avoid distal‐care, Western populations being falsely used as benchmarks for typical socio‐emotional development. Further research across more diverse settings is needed to identify which specific features of infants' caregiving environment—including interactional practices, household and caregiving structure, material conditions, and broader socio‐cultural experiences—contribute to variation in responses to the still‐face paradigm and emotional development more generally.

## Author Contributions

**Carlo Vreden:** conceptualization, formal analysis, investigation, methodology, project administration, visualization, writing – original draft, writing – review and editing. **Eunice Ndyareeba:** methodology, project administration, writing – review and editing. **Herbert E. Ainamani:** writing – review and editing, project administration. **Rebecca Crowther:** investigation, project administration, writing – review and editing. **Helen Donnelly:** investigation, writing – review and editing, project administration. **Beatrice Forward:** investigation, project administration, writing – review and editing. **Brenda Kakai:** investigation, methodology, project administration, writing – review and editing. **Sarah Mazari:** investigation, project administration, writing – review and editing. **Josephine Paricia:** methodology, investigation, writing – review and editing. **Elizabeth Renner:** conceptualization, methodology, investigation, project administration, writing – review and editing. **John Sajabi:** methodology, investigation, writing – review and editing. **Katie Slocombe:** supervision, writing – review and editing. **Georgia Tuohy:** investigation, project administration, writing – review and editing. **Zanna Clay:** conceptualization, methodology, funding acquisition, project administration, resources, supervision, writing – original draft, writing – review and editing.

## Conflicts of Interest

The authors declare no conflicts of interest.

## Supporting information


Supporting Information S1

## Data Availability

All relevant data and analysis code are available for free and open access on the Open Science Framework: https://osf.io/4chvy/?view_only=a4f3feb698084f6bbaf058ae28c4f0a5.
