# Supplementary material for: Cultural Variation in Emotional Reactivity and Regulation in Infants From Uganda and the UK
Source: Infancy. 2026 Aug 19;31(4):e70116. doi: 10.1111/infa.70116 (PMC13490753; doi:10.1111/infa.70116)
Supplement: Supplementary file 1 [file INFA-31-0-s001.docx]

**Supplementary Materials**

**S1 Additional Demographic and Contextual Characteristics of Participating Families Across Study Sites**

Table S1 presents descriptive demographic and contextual characteristics of participating families at each study site. These measures were collected to characterise the broader caregiving environments in which infants were developing. Several variables were assessed using locally appropriate measures that were not directly comparable across sites (e.g., household income bands in the UK, inclusion of often underage live-in household help in household size in urban Uganda), and therefore are presented descriptively rather than for formal statistical comparison.

**Table S1 Additional Characteristics Information**

| Characteristic | Rural Uganda | Urban Uganda | UK |
| --- | --- | --- | --- |
| Highest Maternal Education (Primary/Secondary/Tertiary/None) | 71%/19%/5%/4% | 42%/35%/22%/1% | 0%/3%/97%/0% |
| Household Income | Median monthly income = ca. £17 | Median monthly income = ca. £60 | Median annual household income band: £45,000-60,000 per annum |
| Household Internet Access | 19% | 33% | 100% |
| Household TV Ownership | 40% | 73% | 100% |
| Maternal Occupation (Before Maternity Leave) | 91% subsistence farming, 9% formally employed | 42% unemployed, 58% formally employed or small business owner | 92% formally employed or self-employed, 8% not formally employed |
| Primary Caregiver of Participating Infant | 53% mother, 7% other adult, 38% child | 86% mother, 1% father, 6% other adult, 11% child, 5% maid | 91% mother, 9% mother and father equally |
| Household Size (Including Adults, Children, and Participant) | M = 5.48 (range 2-12) | M = 4.28 (range 2-10) | M = 3.69 (range 2-6) |
| Children in Household (Excluding Participant) | M = 2.28 (range 1-8) | M = 1.23 (range 1-7) | M = 0.73 (range 0-3) |

*Note.* Values are presented as percentages or means (with ranges), unless otherwise indicated. Education refers to the highest level of education attended, including incomplete education at that level (e.g., primary = attended at least one year of primary school). Household income was assessed using locally appropriate measures: average monthly (or seasonal for farming families, converted to monthly) income in Uganda and annual household income bands in the UK.

**S2 Behavioural Coding Scheme**

**Table S2 Behavioural Coding Scheme**

| Infant Behaviour | Score | Description |
| --- | --- | --- |
| Positive Affect | 0 | No positive affect, neutral face with facial muscles relaxed or other affective facial expression (e.g., negative affect) |
|  | 1 | Low intensity smiling (corners of mouth go up), no accompanying positive vocalisation |
|  | 2 | Medium intensity smiling (corners of mouth go up, cheeks raised), can be paired with chuckling/giggling (but not full open-mouthed laughter) |
|  | 3 | High intensity smile paired with open-mouthed laughing vocalisation |
| Negative Affect | 0 | No negative affect, neutral face with facial muscles relaxed or other affective facial expression (e.g., positive affect) |
|  | 1 | Low intensity negative affect including grimacing/frowning (corners of mouth go down), furrowed brows, no accompanying vocalisation |
|  | 2 | Medium intensity negative affect including grimacing/frowning paired with whining/whimpering/fussing or shorter cry/protest yell vocalisation of <2s |
|  | 3 | High intensity negative open-mouthed continuous crying for >2s |
| Gaze | 0 | No eye contact, not looking at the mother’s eyes/face |
|  | 1 | Looking at the mother’s eyes/face for less than half of the time bin (30s play and reunion phases, 15s still-face phase) |
|  | 2 | Looking at the mother’s eyes/face for more than half of the time bin with gaze version lasting |
|  | 3 | Continuous looking at the mother’s eyes/face with maximum look-aways of <1s |

**S3 Full Model Estimates**

Where a reduced model (no interaction terms) was used to assess main effects of fixed effect variables (i.e., an interaction was not significant), the model estimates of this reduced model, not the full model, are presented. Binary variables were dummy coded, with the reference category indicated in parentheses.

**Table S3.1 Positive Affect (Reduced Model)**

| Term | Estimate | SE | Lower CI | Upper CI | p |
| --- | --- | --- | --- | --- | --- |
| Phase (still-face) | -3.61 | 0.43 | -4.45 | -2.77 | <.001 |
| Site (urban Uganda) | -0.25 | 0.39 | -1.00 | 0.51 | .525 |
| Site (UK) | -0.02 | 0.41 | -0.83 | 0.79 | .967 |
| Setup (tested on mat) | -0.26 | 0.36 | -0.96 | 0.45 | .474 |

**Table S3.2 Negative Affect (Full Model)**

| Term | Estimate | SE | Lower CI | Upper CI | p |
| --- | --- | --- | --- | --- | --- |
| Phase (still-face) | 0.17 | 0.44 | -0.70 | 1.04 | .697 |
| Site (urban Uganda) | -0.64 | 0.59 | -1.80 | 0.51 | .276 |
| Site (UK) | -1.24 | 0.64 | -2.49 | 0.00 | .051 |
| Setup (tested on mat) | -1.21 | 0.49 | -2.14 | -0.27 | .012 |
| Phase (still-face) * site (urban Uganda) | 0.89 | 0.66 | -0.40 | 2.18 | .174 |
| Phase (still-face) * site (UK) | 1.90 | 0.69 | 0.55 | 3.24 | .006 |

**Table S3.3 Gaze Affect (Full Model)**

| Term | Estimate | SE | Lower CI | Upper CI | p |
| --- | --- | --- | --- | --- | --- |
| Phase (still-face) | -8.15 | 1.20 | -10.51 | -5.80 | <.001 |
| Site (urban Uganda) | 1.26 | 2.19 | -3.04 | 5.56 | .565 |
| Site (UK) | 11.57 | 2.81 | 6.07 | 17.08 | <.001 |
| Setup (tested on mat) | -0.86 | 1.60 | -4.00 | 2.29 | .593 |
| Phase (still-face) * site (urban Uganda) | -1.14 | 1.93 | -4.92 | 2.64 | .555 |
| Phase (still-face) * site (UK) | -7.72 | 2.49 | -12.60 | -2.85 | .002 |
